# Verapamil Protects against Cartilage Degradation in Osteoarthritis by Inhibiting Wnt/β-Catenin Signaling

**DOI:** 10.1371/journal.pone.0092699

**Published:** 2014-03-21

**Authors:** Akira Takamatsu, Bisei Ohkawara, Mikako Ito, Akio Masuda, Tadahiro Sakai, Naoki Ishiguro, Kinji Ohno

**Affiliations:** 1 Division of Neurogenetics, Center for Neurological Diseases and Cancer, Nagoya University Graduate School of Medicine, Nagoya, Japan; 2 Department of Orthopaedic Surgery, Nagoya University Graduate School of Medicine, Nagoya, Japan; University of Kentucky, United States of America

## Abstract

In past years, the canonical Wnt/β-catenin signaling pathway has emerged as a critical regulator of cartilage development and homeostasis. FRZB, a soluble antagonist of Wnt signaling, has been studied in osteoarthritis (OA) animal models and OA patients as a modulator of Wnt signaling. We screened for FDA-approved drugs that induce *FRZB* expression and suppress Wnt/β-catenin signaling. We found that verapamil, a widely prescribed L-type calcium channel blocker, elevated FRZB expression and suppressed Wnt/β-catenin signaling in human OA chondrocytes. Expression and nuclear translocation of β-catenin was attenuated by verapamil in OA chondrocytes. Lack of the verapamil effects in LiCl-treated and FRZB-downregulated OA chondrocytes also suggested that verpamil suppressed Wnt signaling by inducing FRZB. Verapamil enhanced gene expressions of chondrogenic markers of *ACAN* encoding aggrecan, *COL2A1* encoding collagen type II α1, and *SOX9*, and suppressed Wnt-responsive *AXIN2* and *MMP3* in human OA chondrocytes. Verapamil ameliorated Wnt3A-induced proteoglycan loss in chondrogenically differentiated ATDC5 cells. Verapamil inhibited hypertrophic differentiation of chondrocytes in the explant culture of mouse tibiae. Intraarticular injection of verapamil inhibited OA progression as well as nuclear localizations of β-catenin in a rat OA model. We propose that verapamil holds promise as a potent therapeutic agent for OA by upregulating FRZB and subsequently downregulating Wnt/β-catenin signaling.

## Introduction

Osteoarthritis (OA) is a progressively degenerative joint disorder characterized by degradation of extracellular matrix (ECM) molecules, loss of articular cartilages, and formation of osteophytes. OA causes chronic disability in elderly people and is one of the major health problems worldwide [1]. No rational medical therapy is currently available for OA except for palliative pain control and physiotherapy, before patients require prosthetic joint replacement therapy.

In OA, dysfunction of articular chondrocytes compromises synthesis of ECM and enhances degradation of ECM, which leads to loss of ECM and cartilage degradation. Aggrecan (ACAN) is the most prominent proteoglycan in cartilage, which holds a large amount of water and ions, and confers mechanical elasticity. Collagens, and in particular collagen II, constitute a highly structured fibrillar network that holds spatial arrangement of tissue and provides tensile strength. ECM including ACAN and collagens are degraded by matrix metalloproteinases (MMPs), aggrecanases (ADAMTSs), and other matrix proteases [2], [3]. Chondrocytes orchestrate fine-tuned gene expressions of ECM molecules and their catabolic enzymes to achieve tolerance to mechanical stress as well as elasticity of articular cartilages, which is compromised in OA.

Recent studies revealed that bone morphogenetic proteins (BMPs) [4], Indian hedgehog (IHH) [5], the hypoxia-induced signaling [6], and the Wnt/β-catenin signaling [7]–[9] induce hypertrophic differentiation of chondrocytes and the subsequent aggravation of OA. Indeed, conditional activation of β-catenin in articular chondrocytes in adult mice leads to premature chondrocyte differentiation and development of an OA-like phenotype [10]. In addition, β-catenin stimulates activity of the ECM catabolic enzymes in articular chondrocytes [11]. We thus sought for a modality to suppress Wnt/β-catenin signaling as a potential therapeutic target for OA.

Extracellular antagonists of Wnt, such as the secreted frizzled-related proteins (SFRPs), the dickkopfs (DKKs), and sclerostin (SOST), have been studied in model animals and patients with OA [12]–[14]. One of the SFRPs, FRZB (also known as SFRP3), is a Wnt antagonist originally identified as a chondrogenic factor in articular cartilage extracts [15]. An amino acid-substituting single nucleotide polymorphism (SNP) in *FRZB* is associated with hip OA [16]. *Frzb*-knockout mice are susceptible to a collagenase-induced OA[12]. FRZB functions as a natural brake on hypertrophic differentiation of articular cartilage [17]. FRZB is thus expected to serve as a potential therapeutic mediator against OA progression.

Drug repositioning strategy, in which a drug already used for a specific disease is applied to treat another disease, has been gaining increased attention from both academia and industry in recent years [18], [19]. The advantage of this strategy is that the identified drugs can be readily applied to clinical practice, because the optimal doses, adverse effects, and contraindications are already established.

Here, we screened FDA-approved compounds to identify a clinically applicable drug that induces *FRZB* gene expression and downregulates Wnt/β-catenin signaling. We identified that one of calcium channel blockers, verapamil, but not other blockers upregulates expression of the *FRZB* gene and induces chondrogenic markers. In chondrogenically differentiated ATDC5 and explants of mouse tibiae, verapamil blocks nuclear localization of β-catenin and induces proteoglycan production. Finally, articular injection of verapamil ameliorated cartilage damages in OA-induced knees in rat model.

## Materials and Methods

### Screening of FDA-approved drugs with dual luciferase reporter assay

The *FRZB* promoter region (positions −2,218 to −1 immediately upstream of the ATG translation start site) was amplified with primers, 5′- ccgCTCGAGTGTAGACCAGGCAAAGTTTGTA-3′ and 5′- ggaAGATCTGGATCTGGGAGCTTCTCCTC-3′, from human genomic DNA isolated from HEK293 cells, in which the 5′ overhangs are indicated by lower case letters and the XhoI and BglII sites are underlined. The amplicon was cloned into pGL4.10-luc2 luciferase vector (Promega) using XhoI and BglII sites (pGL4.10-*FRZB*). Human chondrosarcoma (HCS-2/8) cells were kindly provided by Dr. Masaharu Takigawa at Okayama University [20], and were cultured in Dulbecco's Modified Eagle's Medium (DMEM, Invitrogen) supplemented with 10% fetal bovine serum (FBS, Thermo Scientific). For the *FRZB* promoter assay, ∼1×10^4^ cells were seeded in 96-well culture plates (Falcon) and were transfected with the pGL4.10-*FRZB* as well as phRL-TK encoding Renilla luciferase (Promega) using Fugene6 (Invitrogen) according to the manufacturer's protocols. For measuring β-catenin-mediated transcriptional activation, HCS-2/8 cells were transfected with TOPFlash firefly luciferase reporter vector (M50 Super 8× TOPFlash plasmid, Addgene) and phRL-TK. At 24 hours after transfection, the cells were incubated for 24 additional hours in the presence of 10 μM of 1,186 FDA-approved chemical compounds (Prestwick Chemical). Luciferase activity was measured using the Dual Luciferase Reporter Assay System (Promega) and PowerScan4 (DS Parma Biomedical). Firefly luciferase activity was normalized by Renilla luciferase activity and relative luciferase units (RLU) are indicted.

### Total RNA extraction and real-time RT-PCR analysis

The human studies including using HCS-2/8 cells were approved by the Ethical Review Committee of the Nagoya University Graduate School of Medicine. Human osteoarthritic chondrocyte (OAC) cells were obtained from patients who underwent joint replacement for knee OA after appropriate written informed consent was obtained. The conditioned medium enriched with mouse Wnt3A (Wnt3A-CM) was harvested from L Wnt-3A cells (CRL-2647, ATCC) that stably expressed mouse Wnt3a. OAC cells were treated with variable combinations of verapamil, recombinant human Wnt3A (R&D systems), Wnt3A-CM, and 20 mM LiCl. Total RNA was isolated from OAC cells using Trizol (Life Technologies). The first strand cDNA was synthesized with ReverTra Ace (Toyobo). We quantified mRNAs for *FRZB, ACAN*, *COL2A1*, *SOX9*, *AXIN2*, and *MMP3* using LightCycler 480 Real-Time PCR (Roche) and SYBR Green (Takara). The mRNA levels were normalized for that of *GAPDH*.

### Knockdown of *FRZB* using siRNA

OAC cells were transfected with siFRZB-1 (5′- GGCUAAAGUUAAAGAGAUA -3′and 5′-UAUCUCUUUAACUUUAGCC-3′) or siFRZB-2 (5′- GGAUCGACUCGGUAAAAAA -3′ and 5′-UUUUUUACCGAGUCGAUCC -3′) using Lipofectamine 2000 (Life Technologies) according to the manufacturer's protocols. Four hours after transfection, the medium was changed to fresh DMEM with 10% FBS, and cells were treated with Wnt3A-CM and 25 μM of verapamil.

### Western blotting

OAC cells were treated with Wnt3A-CM as well as with 0, 2.5, 5, 10, and 25 μM of verapamil. After 72 hours, cells were lysed in the ice-cold RIPA Lysis Buffer (Santa Cruz) supplemented with 0.1 mM dithiothreitol, 1 μg/ml leupeptin, 1 mM phenylmethylsulfonyl fluoride, and 1 μg/ml aprontinin. Whole cell lysates were separated on SDS-PAGE and transferred to a nitrocellulose membrane followed by immunoblotting with anti-β-catenin (BD Transduction Laboratories) and anti-β-actin (C4, Santa Cruz) antibodies.

### Alcian blue staining

ATDC5 cells were supplied by the Riken BioResource Center (Tsukuba, Japan). ATDC5 cells were cultured in DMEM/F12 (a mixture of Dulbecco's modified Eagle's medium and Ham's F12 medium) (Sigma–Aldrich) supplemented with 5% fetal bovine serum (FBS, Thermo Scientific). ATDC5 cells were differentiated into chondrocytes with insulin-transferrin-selenite (ITS, Invitrogen) for two weeks, and were treated with Wnt3A-CM as well as with 0, 2.5, 5, 10, and 25 μM verapamil. After 72 hours, cells were fixed with methanol for 30 minutes at −20°C, and stained overnight with 0.5% Alcian Blue 8 GX (Sigma) in 1N HCl. For quantitative analyses, Alcian blue-stained cells were lysed in 200 μl of 6 M guanidine HCl for six hours at room temperature. The optical density of the extracted dye was measured at 630 nm using PowerScan 4 (DS Pharma Biomedical).

### Immunofluorescence staining

OAC were fixed with 4% paraformaldehyde at room temperature. Coronal paraffin sections of proximal tibial growth plates were deparaffinized and hydrated in xylene. The specimens were then treated with a blocking buffer including 2% goat serum in 0.5% Triton-X100 for 60 minutes and incubated with mouse anti-β-catenin antibody (BD Transduction Laboratories, 1∶500 dilutions for the cells and 1∶100 dilutions for the sections) at 4 °C overnight. The specimens were incubated with goat anti-mouse fluorescein isothiocyanate (FITC) secondary antibody (1: 500 dilution) at room temperature for one hour. Finally, the specimens were mounted in VectaShield containing 1.5 μg/ml DAPI (Vector Laboratories, Peter-borough, UK) and visualized using IX71 (Olympus). The ratio of β-catenin-positive cells were automatically estimated by dividing FITC-positive cells by the number of DAPI-positive nuclei using MetaMorph (Molecular Device)

### Explant culture of tibiae of mouse embryo

All animal studies were approved by the Animal Care and Use Committee of the Nagoya University and the animals were sacrificed under deep anesthesia, if necessary. maintained according to the guidelines for the care of laboratory animals of the Gifu International Institute of Biotechnology. Tibiae of wild type mouse embryo (E17.5) were dissected under the microscope, placed in a 24-well plate, and cultured in α-minimal essential medium (Invitrogen) supplemented with 0.2% bovine serum albumin, 1 mM β-glycerophosphate, and 50 μg/ml ascorbic acid. Embryonic tibiae were further treated with 20 mM LiCl or with 0, 25, and 50 μM verapamil for 10 days, then fixed in 10% formaldehyde in phosphate-buffered saline, demineralized with 0.5 M EDTA, and embedded in paraffin. Sections were stained with hematoxylin-eosin and Alcian blue. Hypertrophic cells were defined as having a length along the longitudinal axis greater than 10 μm under light microscopy. The numbers of hypertrophic chondrocytes were counted by two blinded observers and averaged.

### *In vivo* generation of OA and its morphological evaluation

Wistar/ST rats were deeply anesthetized with an intraperitoneal injection of pentobarbital sodium (10 mg/ml×0.1 ml). Under sterile conditions, the right knee was induced to osteoarthritis by resection of the menisco-tibial ligament to destabilize medial meniscus (DMM surgery). Verapamil (50 μM) in 50 μl PBS was intraarticularly injected into the right knee each week. The skin and joint capsule on the left knee was incised (sham side). At four and eight weeks postoperatively, rats were sacrificed and tissue around the knees was fixed overnight in 4% paraformaldehyde at 4 °C, dehydrated, and embedded in paraffin. The sagittal sections were stained with Safranin O and Fast-green. OA progressions were graded according to the modified Mankin histologic score on both tibial and femoral sides of articular cartilages [21], [22]. The modified Mankin score was a sum of the following seven parameters: articular cartilage structure, grades 0–11; tidemark duplication, grades 0–3; Safranin O staining, grades 0–8; fibro-cartilage, grades 0–2; chondrocyte clones in uncalcified cartilage, grades 0–2; hypertrophic chondrocytes in calcified cartilage, grades 0–2; and subchondral bone, grades 0–2. The grades of OA were estimated by two blinded observers and averaged.

### Screening of verapamil-activated signaling pathways that facilitate upregulation of *FRZB*

For screening for involvement of other signaling molecules in verapamil-mediated activation of FRZB promoter, HCS-2/8 cells were co-transfected with the pGL4.10-*FRZB* and pCMV-SPORT6 Smurf1 (IMAGE clone 3660965, DNAFORM) or pCMV-SPORT6 Smurf2 (IMAGE clone 5345689, DNAFORM) and phRL-TK. At 24 hours after transfection, the cells were incubated for 24 additional hours in the presence of 50 μM of Verapamil with specific inhibitors of other signaling, SP600125 (JNK inhibitor), PD98059 (Erk inhibitor). Luciferase activity was measured using the Dual Luciferase Reporter Assay System (Promega)

### Statistical analysis

Data are presented as the mean ± SEM. Statistical significance was determined either by unpaired t-test or one-way ANOVA followed by Tukey's post-hoc test. The Jonckheere-Terpstra trend test was used to assess dose responses, and is indicated by a letter ‘trend’ followed by a *p* value. Although one-way ANOVA gave more stringent values than the Jonckheere-Terpstra trend test, one-way ANOVA was not suitable for estimating dose responses. *P*-values less than 0.05 were considered significant. The statistical analyses were performed with SPSS Statistics 21 (IBM).

## Results

### Verapamil enhances the *FRZB* promoter activity and reduces Wnt/β-catenin signaling activity

**Figure 1:**
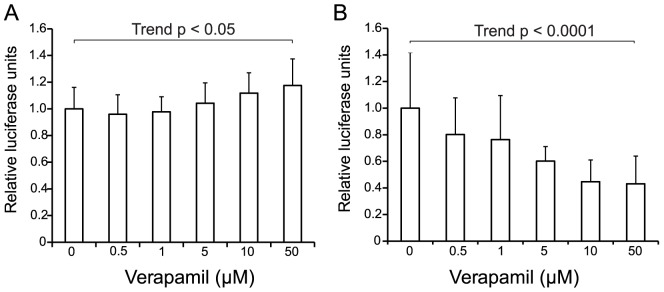
Verapamil activates *FRZB* promoter and reduces Wnt/β-catenin signaling. Luciferase activities in human chondrosarcoma (HCS) cells treated with the indicated concentrations of verapamil for 24 hrs. Firefly luciferase activity for *FRZB* promoter (A) or TOPFlash reporter activity (B) are normalized by the TK promoter-driven Renilla luciferase activity and expressed as relative luciferase units. The mean and SEM (*n* = 12) are indicated. ** *p*<0.01 and *p<0.05 versus control by one-way ANOVA with Tukey's test.

To identify a clinically applicable compound for OA, we transfected HCS-2/8 cells with either pGL4.10-*FRZB* to estimate the *FRZB* promoter activity or TOPFlash to estimate the Wnt/β-catenin activity. We searched for a compound that enhances the *FRZB* promoter and suppresses the Wnt/β-catenin activity among FDA-approved chemical compounds (Prestwick Chemical). These assays were repeated three times and we chose 41 best compounds. With the 41 compounds, we further repeated the assays three additional times. After the first and second rounds of screening, we chose 18 best compounds that consistently exhibited beneficial effects (data not shown). We next examined the dose-dependence by adding 0.5, 1, 5, 10, 50 μM of the 18 compounds. Among them, verapamil, a calcium channel blockers, showed the most consistent and promising dose-dependent activation of the *FRZB* promoter activity and can be used for a long time without major adverse effects (Figure 1A). We also found that verapamil suppressed the Wnt/β-catenin activity in a dose-dependent manner (Figure 1B). Verapamil is an L-type calcium channel blocker that has long been used for hypertension, angina pectoris, cardiac arrhythmia, and most recently cluster headache [23]. We also examined seven other calcium channel blockers (nifedipine, thioridazine, diltiazem, loperamide, perhexiline, nicardipine, felopdipine), but none had an effect (data not shown).

### Verapamil upregulates native FRZB and induces expressions of chondrogenic genes in osteoarthritic chondrocyte (OAC) cells

**Figure 2:**
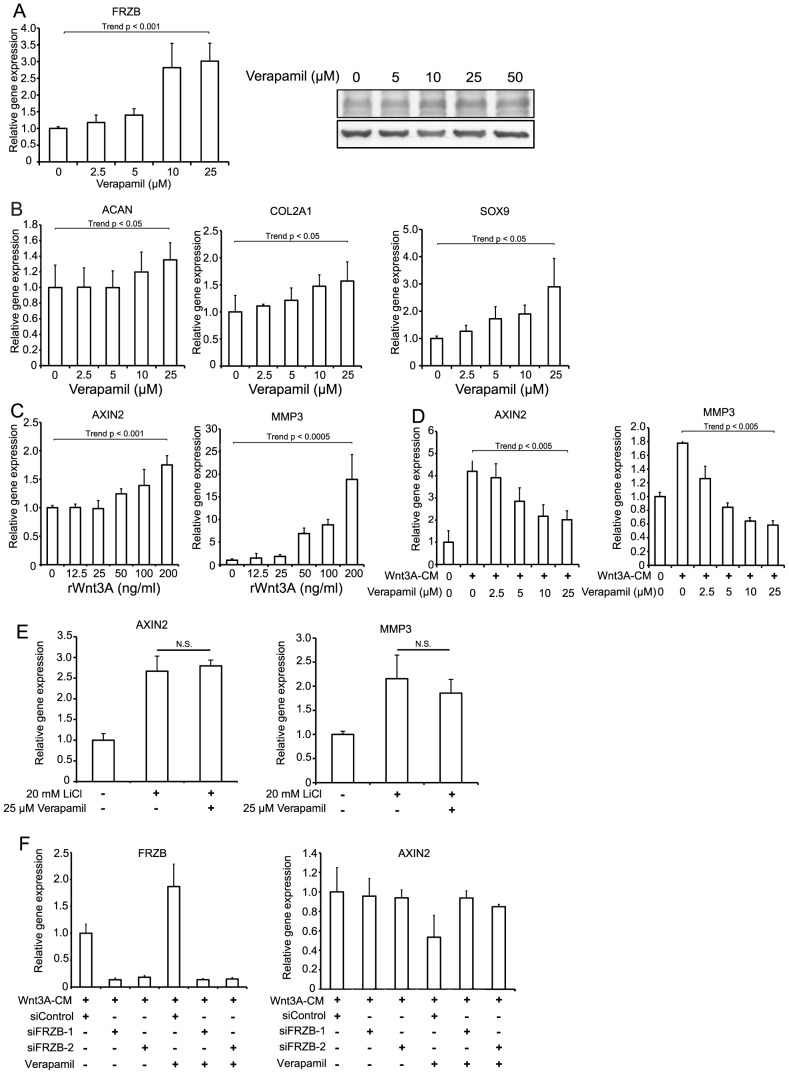
Verapamil upregulates chondrogenic makers (*ACAN* encoding aggrecan, *COL2A1*, and *SOX9*) and downregulates Wnt-responsive *AXIN2* and *MMP3* in human osteoarthritic chondrocytes (OAC) cells. Expression levels of each mRNA are normalized to that without treatment. (A) Verapamil upregulates the native *FRZB* at the mRNA and protein levels in OAC cells (*n* = 3). (B) Gene expressions of *ACAN*, *COL2A1*, and *SOX9* are increased by verapamil in 24 hrs. Purified Wnt3A protein increases *AXIN2* and *MMP3* mRNA expressions in 24 hrs (C), and verapamil inhibits the Wnt3A-induced gene expressions (D). (E) Verapamil does not suppress LiCl-induced *AXIN2* and *MMP3* expressions. (F) *FRZB* siRNAs (siFRZB-1 and siFRZB-2) cancel the effects of verapamil in Wnt3A-treated OAC cells. The mean and SEM (*n* = 3) are indicated. *p<0.05 versus control by one-way ANOVA with Tukey's test.

In OA, breakdown of the extracellular matrix around chondrocytes leads to progressive destruction of articular structures [24]. To investigate the effect of verapamil on human OAC cells, we isolated OAC cells from patients with severe OA undergoing total knee replacement surgery. We first confirmed that verapamil upregulated the native FRZB at the mRNA and protein levels (Figure 2A). We screened signaling pathways that were potentially activated by verapamil to facilitate upregulation of FRZB, but found none (Figure S2).

**Figure 3:**
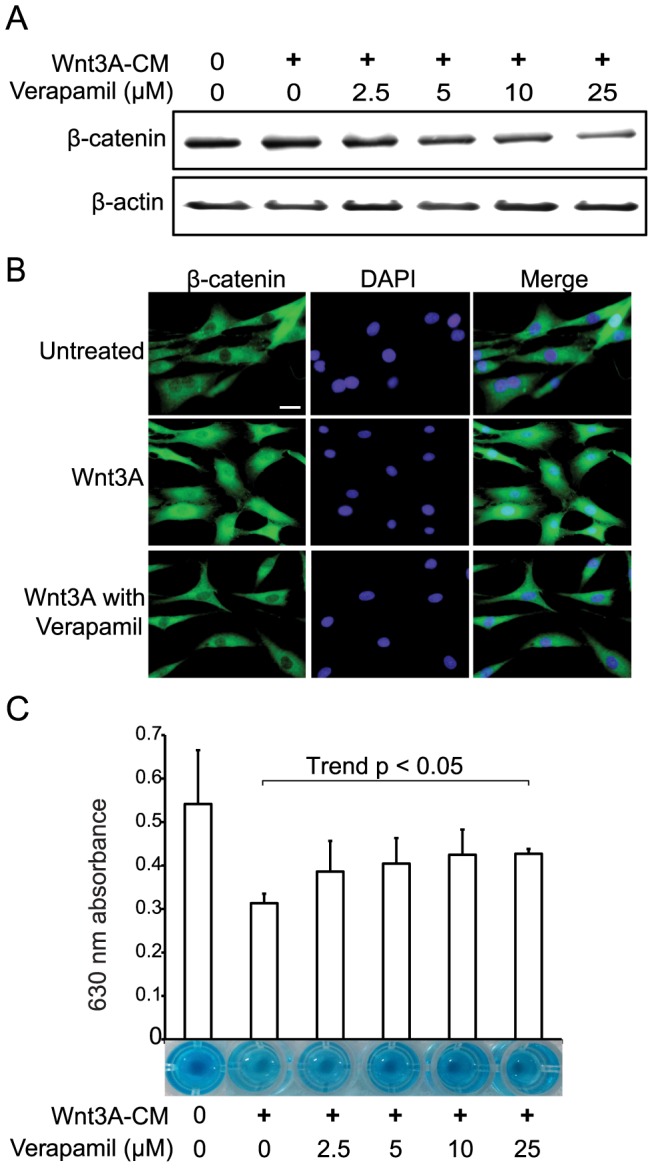
Verapamil suppresses Wnt-mediated protein expression and nuclear translocation of β-catenin in human osteoarthritic chondrocytes (OAC) cells, and verapamil rescues Wnt-induced loss of proteoglycans in chondrogenically differentiated ATDC5 cells. (A) Immunoblotting of β-catenin in OAC cells treated with Wnt3A-CM and verapamil for 72 hrs. (B) Immunofluorescence staining with anti-β-catenin antibody in OAC cells. Untreated cells (upper panels), cells treated with Wnt3A-CM alone (middle panels), and cells treated with Wnt3A-CM and 25 μM verapamil (lower panels) for 72 hours are stained with anti-β-catenin antibody (green, left panels) and DAPI (blue, middle panels). Wnt3A-induced nuclear localization of β-catenin is blocked by verapamil. Scale bar = 20 μm. (C) Alcian blue staining of ATDC5 cells that are differentiated to chondrocytes with ITS for two weeks. The cells are subsequently treated with Wnt3A-CM and verapamil for 72 hrs. Proteoglycans are quantified by measuring the optical density at 630 nm of the cell lysates. The mean and SEM (*n*  = 3) are indicated. *p<0.05 versus control by one-way ANOVA with Tukey's test.

Verapamil also upregulated mRNAs for *ACAN* encoding aggrecan, *COL2A1* encoding collagen type II α1, and *SOX9* encoding SRY-box 9 in a dose-dependent manner (Figure 2B). We next confirmed in OAC cells that Wnt3A upregulated *AXIN2* mRNA, a specific marker of Wnt/β-catenin signaling [25] and *MMP3* mRNA, a gene encoding catabolic metalloproteinase 3 (Figure 2C). As expected, verapamil suppressed *AXIN2* and *MMP3* in a dose-dependent manner (Figure 2D). We additionally observed that verapamil suppressed Wnt3A-mediated expression of total cellular β-catenin (Figure 3A), and nuclear translocation of β-catenin (Figure 3B).

LiCl is an inhibitor of Gsk3β and activates Wnt/β-catenin signaling. As expected, verapamil had no effects on LiCl-induced upregulations of *AXIN2* and *MMP3* in OAC cells (Figure 2E), whereas knocking down of *FRZB* cancelled the effects of verapamil (Figure 2F). These results underscored a notion that verapamil upregulates expression of FRZB and downregulates Wnt/β-catenin signaling in OAC cells.

### Verapamil rescues Wnt3A-induced degradation of proteoglycans in differentiated ATDC5 cells

Chondrocytes produce and maintain the cartilaginous matrix, which is mostly comprised of collagens and proteoglycans [7]. To investigate the effects of verapamil on degradation of proteoglycans, we performed Alcian blue staining to quantify acidic polysaccharides, such as glycosaminoglycans, in differentiated mouse chondrogenic ATDC5 cells. As OAC cells were not able to produce an appreciable amount of proteoglycans (data not shown), we used chondrogenically differentiated ATDC5 cells. We found that Wnt3A treatment induced loss of proteoglycans in ATDC5 cells and verapamil rescued the loss in a dose-dependent manner (Figure 3C).

### Verapamil suppresses hypertrophic differentiation of chondrocytes in growth plates

**Figure 4:**
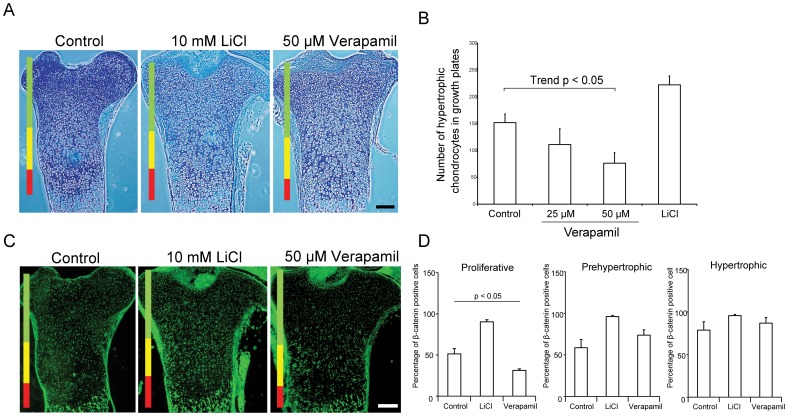
Verapamil suppresses hypertrophic differentiation of chondrocytes and β-catenin staining in growth plates in explanted mouse fetal tibiae on embryonic day 17.5. (A) Tibiae are cultured with LiCl or verapamil for 10 days, and coronal slices of paraffin sections are stained with Alcian blue combined with hematoxylin and eosin staining. Three layers of proliferative (green), prehypertrophic (yellow), and hypertrophic (red) zones are indicated by bars. Scale bar = 200 μm. (B) Verapamil suppresses the number of chondrocytes in the hypertrophic zone in (A). (C) Immunofluorescence with antibody against β-catenin in proximal tibiae of mouse embryo (E17.5). Color bars indicate layers as indicated in (A). Scale bar = 200 μm. (D) Verapamil suppresses the number of β-catenin-positive cells in the proliferative zone. The mean and SEM (*n* = 3) are indicated. *p<0.05 versus control by one-way ANOVA with Tukey's test.

Differentiation of chondrocytes including endochondral ossification is essential for embryonic skeletal growth, which has recently been demonstrated to be abnormally operational in development of OA [26]. Wnt/β-catenin signaling facilitates hypertrophic differentiation of chondrocytes in embryonic growth plates [27]. To investigate the effects of verapamil on hypertrophic differentiation of chondrocytes in growth plates, we cultured explanted mouse fetal tibiae with verapamil. We also treated the explanted tibiae with LiCl to confirm the responsiveness to Wnt/β-catenin. Verapamil had no gross effects on the metaphysis and diaphysis. Zonal analysis of the proximal growth plates showed that LiCl increased and verapamil decreased the height of the hypertrophic zone (Figure 4A). The numbers of hypertrophic chondrocytes counted by two blinded observers also underscored LiCl-mediated enhancement and verapamil-mediated suppression of the hypertrophic zone (Figure 4B). Immunostaining of β-catenin also showed that verapamil inhibited accumulation of β-catenin in growth plates, especially in the proliferative zone (Figures 4C and 4D). These results suggest that verapamil has an inhibitory effect on hypertrophic differentiation of chondrocytes in growth plates.

### Verapamil ameliorates OA in a rat model

ure 5C). These results indicate that verapamil inhibits Wnt signaling and ameliorates progression of OA *in vivo*.

## Discussion

**Figure 5:**
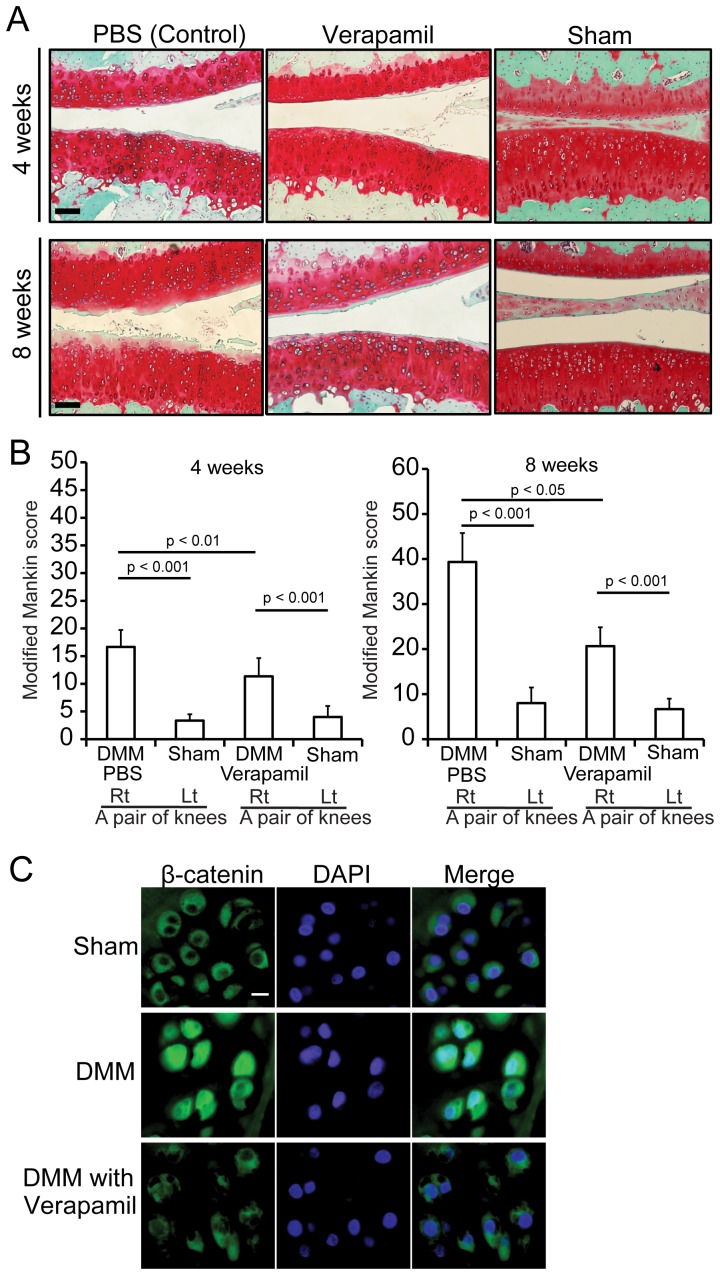
Verapamil prevents OA progression and β-catenin accumulation in rat DMM model. **(A, B)** DMM surgery induces mild OA phenotype in Wistar/ST rat and verapamil prevents OA progression. (A) Representative staining of knee joints with Safranin O and fast green. Scale bars = 200 μm. (B) Verapamil suppresses OA progressions evaluated by modified Mankin score at four and eight weeks after the surgery. Three rats in each group had DMM and sham surgeries in the right and left knees, respectively. Mean and SEM (*n* = 3) are indicated. (C) Immunofluorescence staining with anti-β-catenin antibody in rat articular cartilage. Articular chondrocytes at the weight-bearing sites are stained with anti-β-catenin antibody and DAPI. Nuclear translocation of β-catenin in DMM is blocked by verapamil. Scale bar = 20 μm.

In physiological endochondral ossification, chondrocytes become hypertrophic and remove the extracellular matrix proteins by expressing MMPs and ADMTSs. Chondrocytes finally die by apoptosis and are substituted by osteoblasts [28],[29]. Recent studies disclose that OA follow a similar path to the physiological endochondral ossification: chondrocytes lose the stable phenotype and undergo terminal differentiations, as indicated by upregulation of marker genes for hypertrophy [26]. Wnt/β-catenin signaling pathway is known to drive endochondral ossifications by upregulating MMPs and ADAMTSs [30] in both physiological and pathological conditions [8]. Homeostasis of cartilage in adults is thus maintained by suppressed Wnt/β-catenin signaling, which is exemplified by the fact that FRZB functions as a natural brake to hypertrophic differentiation of chondrocytes [17]. Here we investigated the effects of verapamil on development and progression of OA, and revealed that verapamil (i) enhances *FRZB* gene expression, (ii) inhibits Wnt/β-catenin signaling, (iii) suppresses ECM degradation, (iv) inhibits hypertrophic differentiation of chondrocytes, and (v) ameliorates OA model rats. We used up to 50 μM verapamil *in vitro* (Figure 1), *ex vivo* (Figure 4), and *in vivo* (Figure 5) without overt adverse effects, although the feasibility of intraarticular injection of 50 μM verapamil in clinical practice needs to be carefully validated.

In addition to FRZB, verapamil upregulates another chondrogenic key gene, *SOX9* (Figure 2B). SOX9 is a transcriptional factor that drives chondrogenic differentiation including upregulation of *COL2A1* [31], and is a physiological inhibitor against hypertrophic conversion of chondrocytes in growth plates [32]. Interestingly, physiological binding of SOX9 and β-catenin degrades both SOX9 and β-catenin, which indicates that activated Wnt/β-catenin degrades SOX9 and vice versa [33]. Verapamil is thus expected to increase the amount of SOX9 by suppressing Wnt/β-catenin. This mechanism is likely to account for the verapamil-mediated upregulation of *SOX9* transcripts (Figure 2B), because SOX9 protein upregulates expression of *SOX9* mRNA by forming a positive feedback loop [34].

As stated in the introduction, activation of Wnt/β-catenin worsens OA [7]-[11] and its inhibition ameliorates OA [12]–[14], [17]. In contrast, however, constitutive inhibition of β-catenin in chondrocytes also leads to OA in mice [35]. Additionally, another Wnt antagonist, DKK1, promotes secretion of matrix proteinases in synovial fibroblasts and accelerates cartilage destruction [36]. These reports suggest that excessive suppression of Wnt/β-catenin may be rather deleterious in OA. The intraarticular administration of a high concentration of verapamil was likely to have suppressed Wnt/β-catenin signaling moderately and exerted beneficial effects in our rat model of OA.

The ATP-binding cassette (ABC) transporter exports hyaluronan to the extracellular matrix space [37] and hyaluronan is abnormally overproduced in OA cartilage [38]. As multidrug resistance (MDR) inhibitors including verapamil inhibits the ABC transporter [39], verapamil was intraarticularly injected in a rat OA model and verapamil indeed prevented abnormal production of hyaluronan and loss of aggrecan in osteoarthritic rat knees [40]. We observed a similar effect of verapamil on OA and have shown that the effects are mediated by upregulation of FRZB. Wnt/β-catenin signaling upregulates the ABC transporter in cerebral endothelium [41] and cancerous cells [42]. Although Wnt-mediated upregulation of the ABC transporter has not been reported in chondrocytes to our knowledge, suppression of the ABC transporter is likely to be another target of FRZB. As verapamil was approved as a class IV antiarrhythmic agent by FDA and has long been used without major adverse effects, verapamil holds promise as a therapeutic option for patients suffering from OA.

## Supporting Information

Figure S1 **Representative low magnification images of articular surfaces of rat knees after DMM surgery shown in** **Fig. 5A** **(boxed).** Sections are stained with Safranin O and fast green. Scale bars = 500 μm.
(TIF)

Figure S2 **Firefly luciferase activity for** ***FRZB*** **promoter in HCS cells treated with verapamil alone or in the presense of inhibitor of other signaling molecules.** There are no significant difference between control and each group. Mean and SEM (*n* = 8) are indicated.
(EPS)
