# Supplementary figures and images for: Verapamil Protects against Cartilage Degradation in Osteoarthritis by Inhibiting Wnt/β-Catenin Signaling
Source: PLoS One. 2014 Mar 21;9(3):e92699. doi: 10.1371/journal.pone.0092699 (PMC3962439; doi:10.1371/journal.pone.0092699)

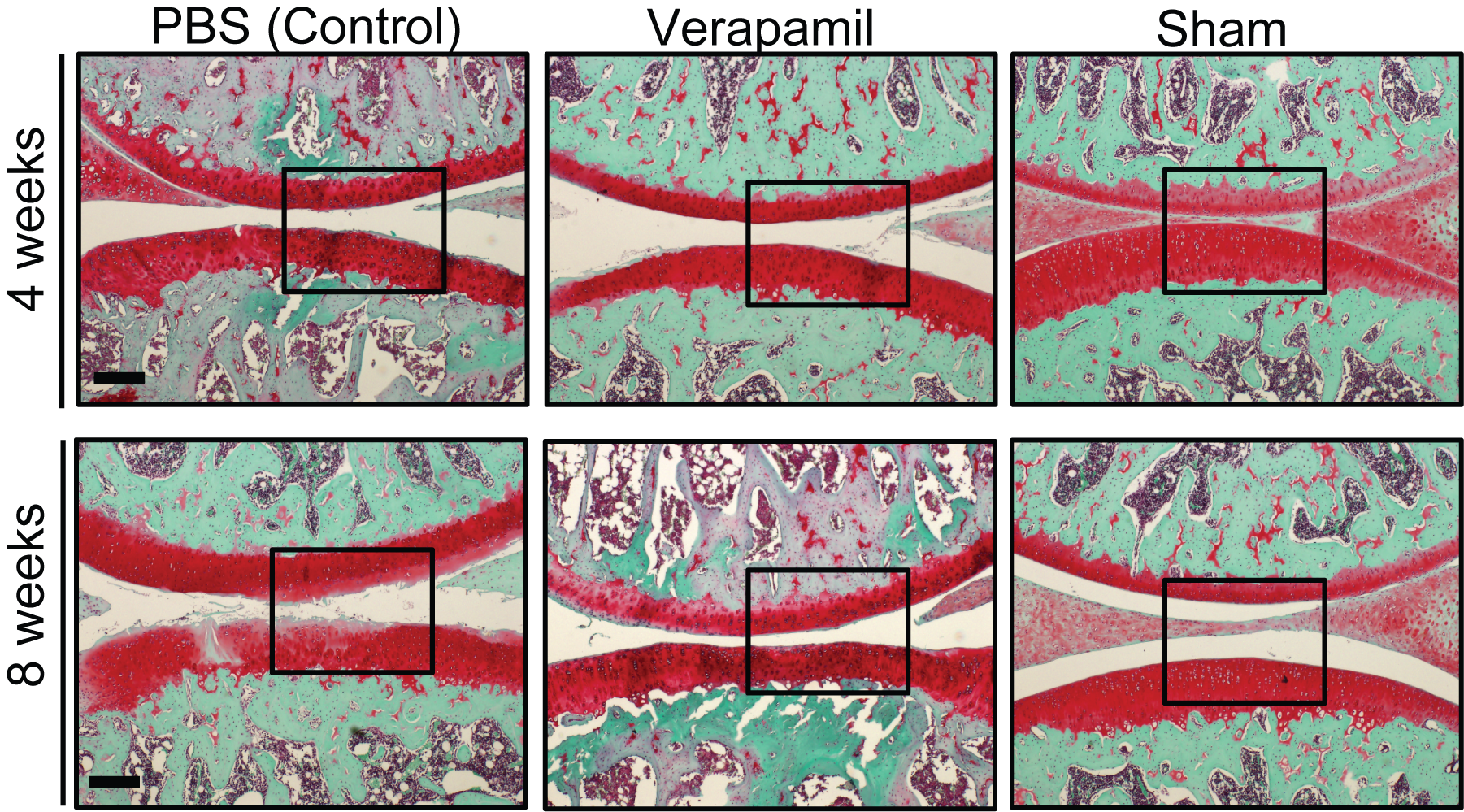

Supplement: Figure S1 — Representative low magnification images of articular surfaces of rat knees after DMM surgery shown in Fig. 5A (boxed). Sections are stained with Safranin O and fast green. Scale bars = 500 μm. (TIF) [file pone.0092699.s001.tif]
